# Resistin-like beta reduction is associated to low survival rate and is downregulated by adjuvant therapy in colorectal cancer patients

**DOI:** 10.1038/s41598-023-28450-1

**Published:** 2023-01-27

**Authors:** Michelino Di Rosa, Antonio Di Cataldo, Giuseppe Broggi, Rosario Caltabiano, Daniele Tibullo, Paola Castrogiovanni, Rosa Imbesi, Raffaele Lanteri, Federico Salomone, Giuseppina Raciti, Giovanni Li Volti

**Affiliations:** 1 Department of Biomedical and Biotechnological Sciences, University of Catania, Via S. Sofia, 97, 95123 Catania, Italy; 2 Department of General Surgery and Medical-Surgical Specialties, University of Catania, Via S. Sofia, 97, 95123 Catania, Italy; 3 Department of Medical and Surgical Sciences and Advanced Technologies “G. F. Ingrassia”, Anatomic Pathology, University of Catania, 95123 Catania, Italy; 4 Division of Gastroenterology, Ospedale di Acireale, Azienda Sanitaria Provinciale di Catania, 95123 Catania, Italy; 5 Department of Drug Sciences, University of Catania, Viale Andrea Doria, 6, Catania, Italy

**Keywords:** Colorectal cancer, Data processing, Databases, Functional clustering, Gene ontology

## Abstract

Colorectal Cancer (CRC) is one of the most common cancers accounting for 1.8 million new cases worldwide every year. Therefore, the identification of new potential therapeutic targets represents a continuous challenge to improve survival and quality of CRC patient’s life. We performed a microarray analysis dataset consisting of colon biopsies of healthy subjects (HS) and CRC patients. These results were further confirmed in a clinical setting evaluating a series of CRC patients to assess the expression of Resistin-Like Beta (*RETNLB*) and to correlate it with their clinical data. Our results showed a significant reduction of *RETNLB* expression in CRC biopsies compared to the HS mucosa. Furthermore, such reduction was significantly associated with the TNM grade and patients’ age. Furthermore, a significantly positive correlation was found within mutated subjects for KRAS, TP53, and BRAF. In particular, patients with poor prognosis at 5 years exhibited *RETNLB* lower levels. In-silico analysis data were confirmed by histochemical analysis in a series of CRC patients recruited by our group. The results obtained provided that *RETNLB* low levels are associated with an unfavorable prognosis in CRC patients and its expression is also dependent on adjuvant therapy. Further studies are warranted in order to evaluate the molecular mechanisms underlying the role of *RETNLB* in CRC progression.

## Introduction

Colorectal cancer (CRC) represents the third cancer type in terms of new cases and is the second most closely related cause of cancer worldwide^1^. The incidence is constantly increasing due to phenomena such as socio-economic developments, pollutants, unbalanced diets and increasing average age^1^. CRC patients will develop 65% of the metastases, of which the most common will be in the liver (approximately 40%). Unfortunately, although new management strategies have been adopted in recent years, including advances in surgical techniques and new adjuvant therapies, mortality from CRC is still high due to postoperative recurrence and metastases^2,3^. Currently, clinical treatment of CRC patients includes curative surgery followed by adjuvant chemotherapy, with protocol variations depending on the disease status^4^. Recently, the rapid development and increased application of next-generation sequencing (NGS) technology allowed to evaluate the molecular profiles of many cancers, including CRC leading to the identification of new molecular biomarkers and potential targets to be exploited for diagnostic and prognostic purposes^5^.

Resistin-like molecules (RELM) are a family of recently identified proteins including RELMα / RETNLA, RELMβ / RETNLB, Resistin/RETN and RELMγ/RETNLG and involved in different disease contexts^6^. Recently, in addition to being implicated in inflammatory processes with microbial etiology, inflammatory diseases and metabolic disorders, some members of this family have been associated with tumor progression^7,8^. Immunocytochemical studies showed that *RETNLB* protein (RELMβ) in localized in the secretory granules of intestinal goblet cells^9^ and is secreted apically in the intestinal lumen^10^. Furthermore, recent studies suggested that *RETNLB* may serve as an immune effector mediating the extrusion of nematodes from the gastrointestinal tract by interfering with their ability to detect the gastrointestinal microenvironment^11^. Finally, *RETNLB* was found to be overexpressed both in a human CRC cell line LS174T^12^, and in a large percentage of CRC patients biopsies (about 80%) positively correlating to patients survival^13^.

The aim of the present study was to investigate the expression and the prognostic value of *RETNLB* in patients with CRC through bioinformatic analysis of dataset from NCBI and The Human Protein Atlas database. We also validated retrospectively such data in a cohort of 44 patients selected from the local University Pathology core collection biopsies.

## Results

### Significant reduction in *RETNLB* expression levels during CRC progression

**Figure 1:**
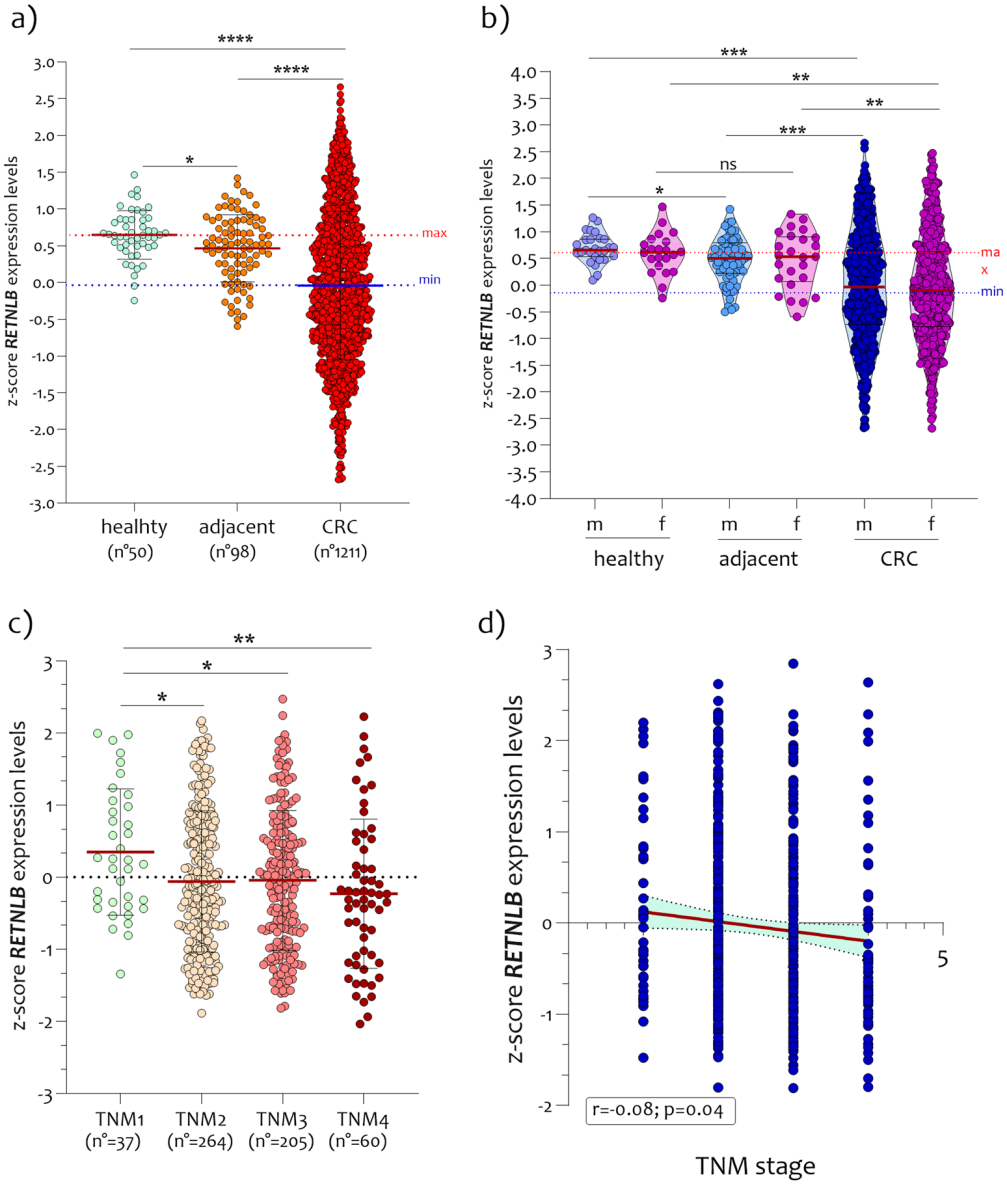
*Expression levels of RETNLB in CRC datasets.* (**a**) Reduction of *RETNLB* expression in CRC biopsies compared to NHM and NPDM; (**b**) Expression analysis of *RETNLB* in CRC biopsies according to the patient’s sex; (**c**) Expression of *RETNLB* in CRC biopsies according to the TNM stage stratification; (**d**) Correlation analysis between the *RETNLB* expression levels and TNM stage in CRC biopsies. The dashed lines indicate the maximum and minimum mean values. Data are expressed as z-score intensity expression levels (means and SD) and presented as dot plots. P values < 0.05 were considered as statistically significant (*p < 0.01; ****p < 0.00001).

**Figure 2:**
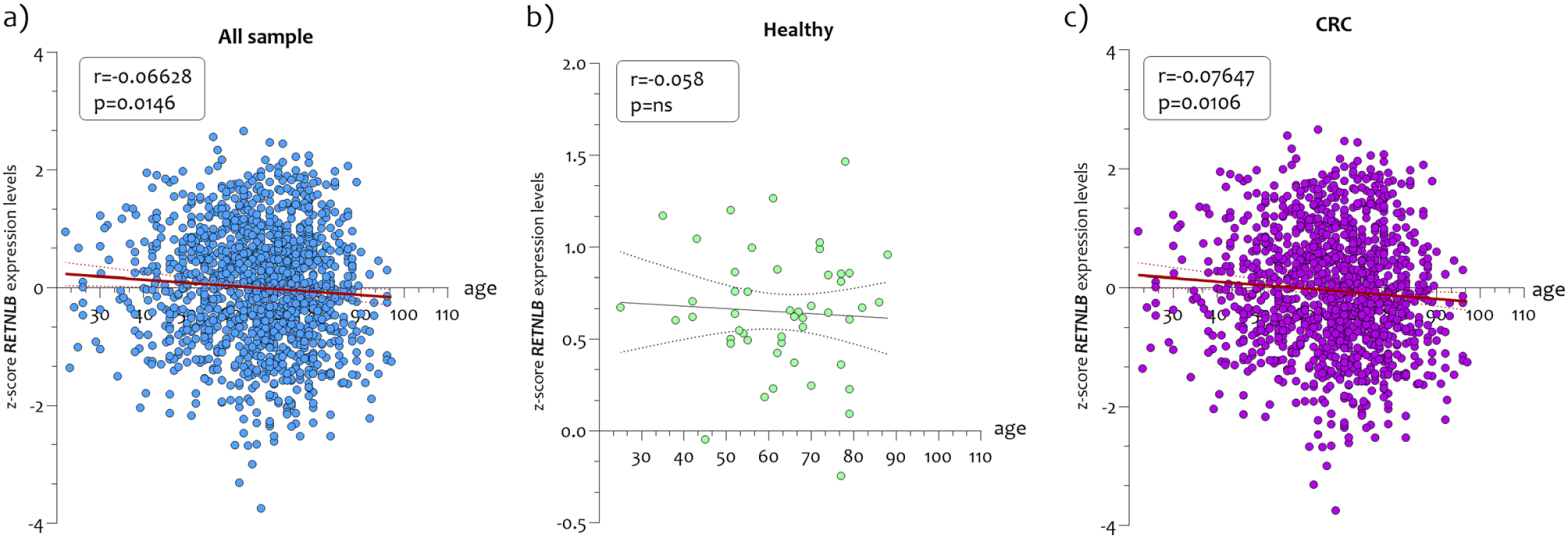
*Inverse correlation between RETNLB levels and age in healthy and CRC patients’ biopsies*. (**a**) Significant inverse correlation between *RETNLB* expression levels and age of all sample analyzed; (**b**) No significant correlation were observed between the *RETNLB* and age in healthy colon mucosa biopsies; (**c**) Significant correlation were observed between the *RETNLB* expression levels and age of patients affected by CRC. The dashed lines indicate the maximum and minimum mean values. Data are expressed as z-score intensity expression levels (means and SD) and presented as dot plots. p values < 0.05 were considered as statistically significant.

In order to analyze *RETNLB* expression levels in biopsies of CRC patients, we merged the datasets after transformation into z-scores. We obtained 50 healthy colon mucosae, 98 non-pathological distant colon mucosa (NPDM) from tumor site, and 1281 CRC biopsies. Expression analysis revealed a significant reduction in *RETNLB* levels both in NPDM (p < 0.01) and in CRC biopsies (p < 0.0001), compared to healthy colon mucosae biopsies (Fig. 1a). Significant variations were highlighted by comparing *RETNLB* expression levels between NPDM and CRC biopsies (p < 0.0001) (Fig. 1a). Differential analysis by sex showed significant variations for both women and men. No intra-sex changes were noted (Fig. 1b). By stratifying the *RETNLB* expression levels obtained from biopsies of CRC patients as a function of the TNM classification, we noted a significantly progressive reduction with increasing TNM grade (p < 0.01 in TNM1 vs TNM2, p < 0.01 in TNM1 vs TNM3, p < 0.001 in TNM1 vs TNM4) (p = 0.04, r = −0.08) (Fig. 1c, d). Furthermore, by stratifying the *RETNLB* expression levels in CRC biopsies patients’ and in healthy subjects according to the age, we showed a significant reduction both considering the whole cohort of samples (p = 0.0146; r = −0.066) and in patients with CRC (p = 0.010; r = −0.076) but not in healthy subjects (p = ns, r = −0.05) (Fig. 2).

### *RETNLB* expression levels are significantly related to TP53, KRAS, and BRAF mutations

**Figure 3:**
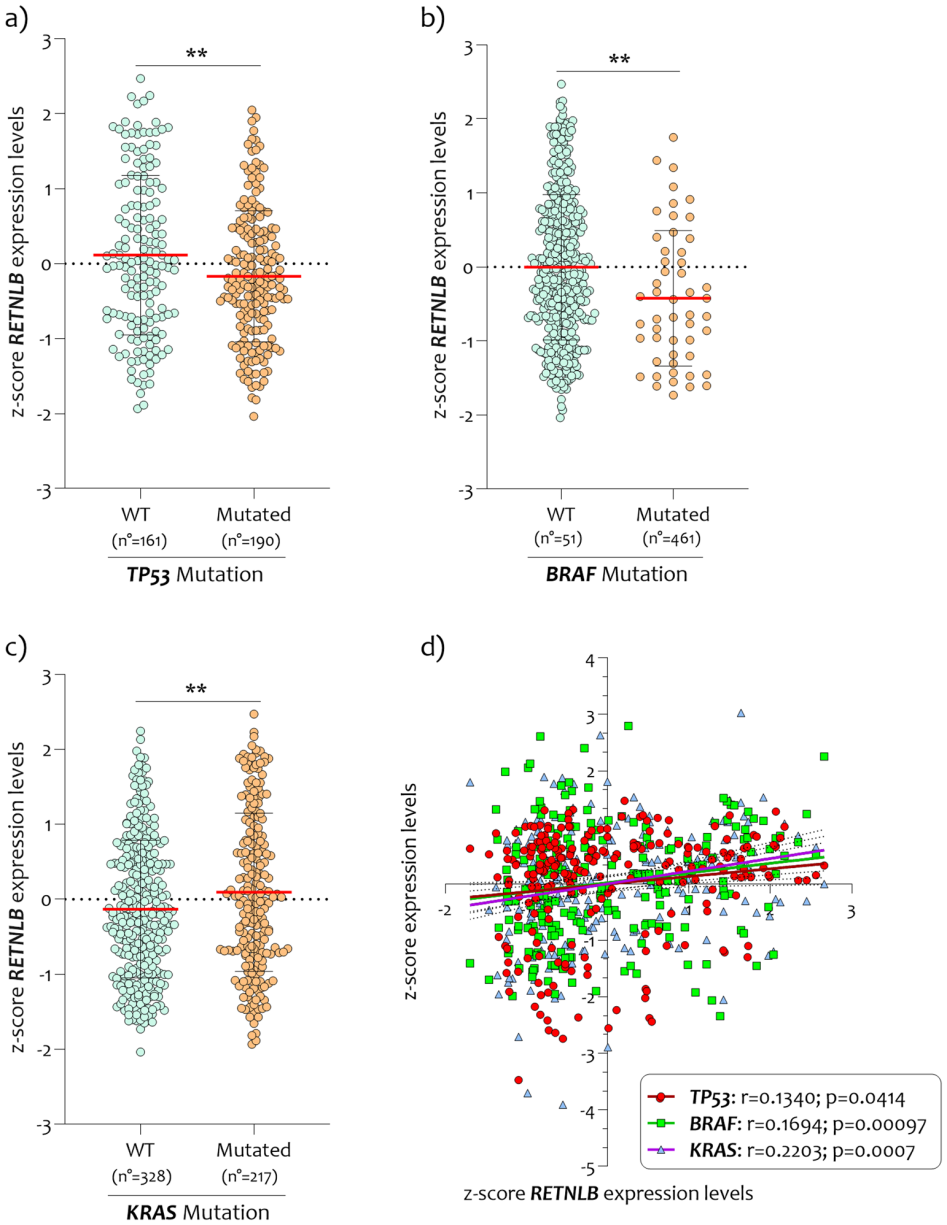
*RETNLB expression levels correlate to BRAF, TP53, and KRAS in CRC biopsies.* (**a**) *RETNLB* expression levels analysis in CRC patients TP53 mutated (n = 190) and wild-type (n = 161); (**b**) *RETNLB* expression levels analysis in CRC patients BRAF mutated (n = 51) and wild-type (n = 461); (**c**) *RETNLB* expression levels analysis in CRC patients KRAS mutated (n = 328) and wild-type (n = 217); (**d**). Data are expressed as z-score intensity expression levels (means and SD) and presented as dot plots. p values < 0.05 were considered as statistically significant (**p < 0.001).

The significant reduction in *RETNLB* expression levels and the inverse correlation with TNM grade prompted us to investigate the possible effect for common DNA alterations, including BRAF, KRAS, and TP53 mutations known to be involved in the CRC pathogenesis. We found that in subjects carrying TP53 and BRAF mutations, *RETNLB* expression levels were significantly reduced compared to wild-types (Fig. 3a, b) and positively correlated with TP53 and BRAF expression levels (Fig. 3d) (p = 0.014, r = 0.1340 and p = 0.00097, r = 0.1694 respectively). Conversely, when we stratified the samples biopsy of CRC patients according to the presence of the KRAS gene mutation, we showed an increase in *RETNLB* expression levels in the mutated subjects compared to wild-type (p < 0.001) (Fig. 3c). The linear regression analysis between *RETNLB* expression levels and KRAS showed positive significance correlation (p = 0.0007, r = 0.2203) (Fig. 3d).

### *RETNLB* is a potential new survival marker in CRC

**Figure 4:**
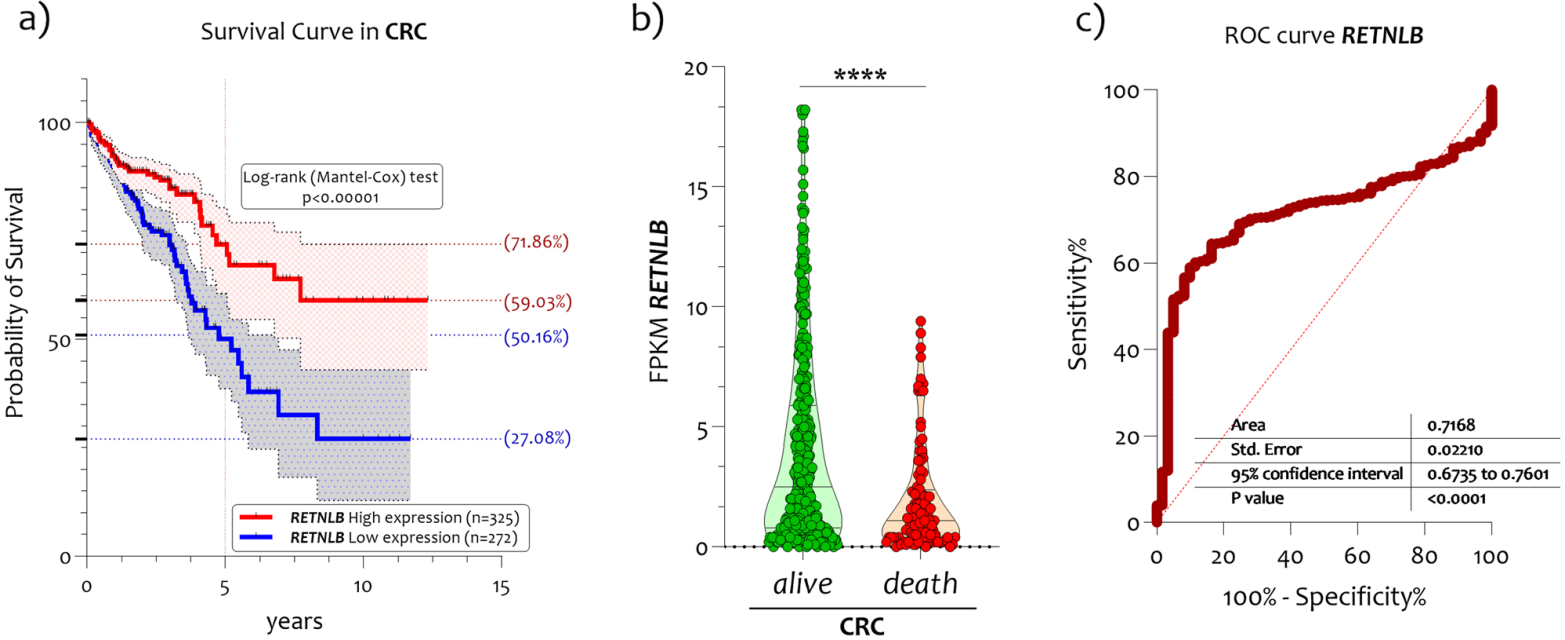
*RETNLB expression levels correlate to the surviving of patients with CRC.* (**a**) Kaplan–Meier plots of correlation between *RETNLB* mRNA expression level and patient survival; (**b**) Expression analysis in CRC patients as a function of 5-year survival; (**c**) High diagnostic ability of *RETNLB* (p < 0.00001, AUC = 0.7168) to discriminate the healthy subjects from CRC patients. The dashed lines indicate the maximum and minimum mean values. Data are expressed as z-score intensity expression levels (means and SD) and presented as dot plots. p values < 0.05 were considered as statistically significant (***p < 0.0001).

**Figure 5:**
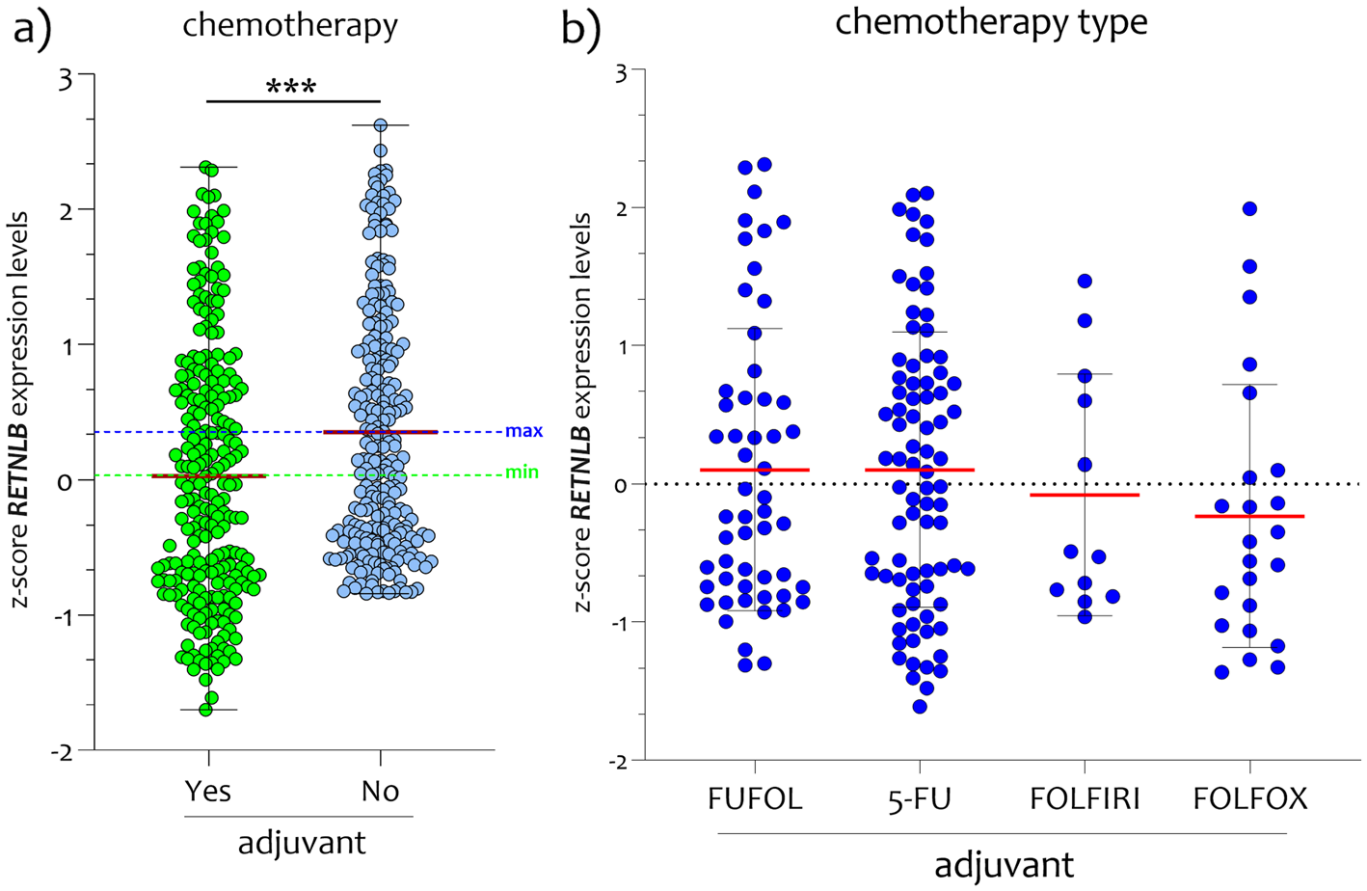
*RETNLB expression levels correlate to treatment of patients with CRC with adjuvant chemotherapy.* (**a**) *RETNLB* levels are reduced in biopsies from CRC patients during adjuvant chemotherapy treatment; (**b**) *RETNLB* expression levels in CRC patients’ biopsies with different adjuvant chemotherapy treatments (5-fluorouracil (5-FU), folinic acid (FUFOL), irinotecan (FOLFIRI), and oxaliplatin (FOLFOX)). The dashed lines indicate the maximum and minimum mean values. Data are expressed as z-score intensity expression levels (means and SD) and presented as dot plots. p values < 0.05 were considered as statistically significant (***p < 0.0001).

In accordance with the obtained results, we downloaded from The Cancer Genome Atlas (TCGA) RNA-seq the data relating to *RETNLB* RNA-seq expression belonging to 597 patients affected by CRC. By establishing as cut-off 2.48 FPKM (number Fragments Per Kilobase of exon per Million reads), we obtained two groups of patients. The first group consisted of patients with *RETNLB* high expression levels (called High expression, n = 325), and a group with low expression levels (named Low expression, n = 272). The prognosis of each group of patients (High and Low) was examined by Kaplan–Meier survival estimators, and the survival outcomes of the two groups were compared by log-rank tests. We found that the two groups separated significantly (p < 0.000001) (Fig. 4a). The 71.86% of CRC patients with high *RETNLB* levels survived at 5 years from disease diagnosis, while for patients with low *RETNLB* levels only 50.16% (Fig. 4a). By separating all patients by survival rate, we found that the deceased had significantly lower levels than those still alive (Fig. 4b). In order to evaluate the potential diagnostic ability of *RETNLB* to discriminate against the CRC patients from healthy subjects, we performed a Receiver operating characteristic (ROC) analysis. We confirmed the high diagnostic ability of *RETNLB* expression levels in colorectal biopsies (p < 0.00001, AUC = 0.7168) to discriminate the healthy subjects from CRC patients (Fig. 4c). Interestingly, when we grouped CRC patients according to adjuvant chemotherapy treatment, we found that *RETNLB* levels were significantly affected by treatment (Fig. 5a). No significant changes were appreciated when comparing *RETNLB* levels during different adjuvant chemotherapy treatments (5-fluorouracil (5-FU), folinic acid (FUFOL), irinotecan (FOLFIRI), and oxaliplatin (FOLFOX)) (Fig. 5b), also by restricting the analysis to the TNM4 stage (data not showed).

## Significant reduction in ***RETNLB*** protein (RELMβ) levels in biopsies of patients with CRC

**Figure 6:**
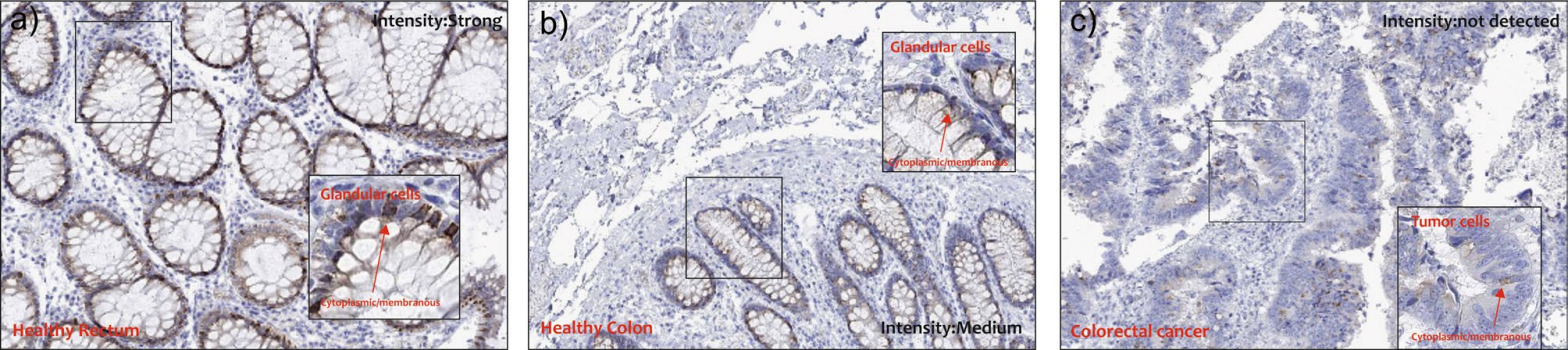
*RETNLB protein detection in healthy rectum/colon and in CRC biopsies from the human atlas protein.* Immunostaining for RELMB in rectum (**a**), colon (**b**), and CRC (**c**). The boxes represent magnification of the original figure. The red arrows indicate the location. The images downloaded from the Human Atlas Project (https://www.proteinatlas.org/) have been re-adapted using CorelDraw. The original files web site links are available as well as the complete analyzes, in Supplementary Table 1.

In order to evaluate the RELMβ proteins expression levels in healthy subjects and CRC patients, we explored the Human Atlas project portal. We selected the histological biopsies of healthy and pathological tissue with protein staining RELMβ. As regards the detection, we observed high-intensity staining of RELMβ in the rectum and medium-intensity staining in healthy colon (Fig. 6 a,b). No detection was observed in CRC biopsies. The immunostaining details are reported in Supplementary Table 1. As regards localization, we reported positive staining in glandular cells with a localization cytoplasmic/membranous (Fig. 6a, b, c).

### Immunohistochemical findings of the cases from our series

**Figure 7:**
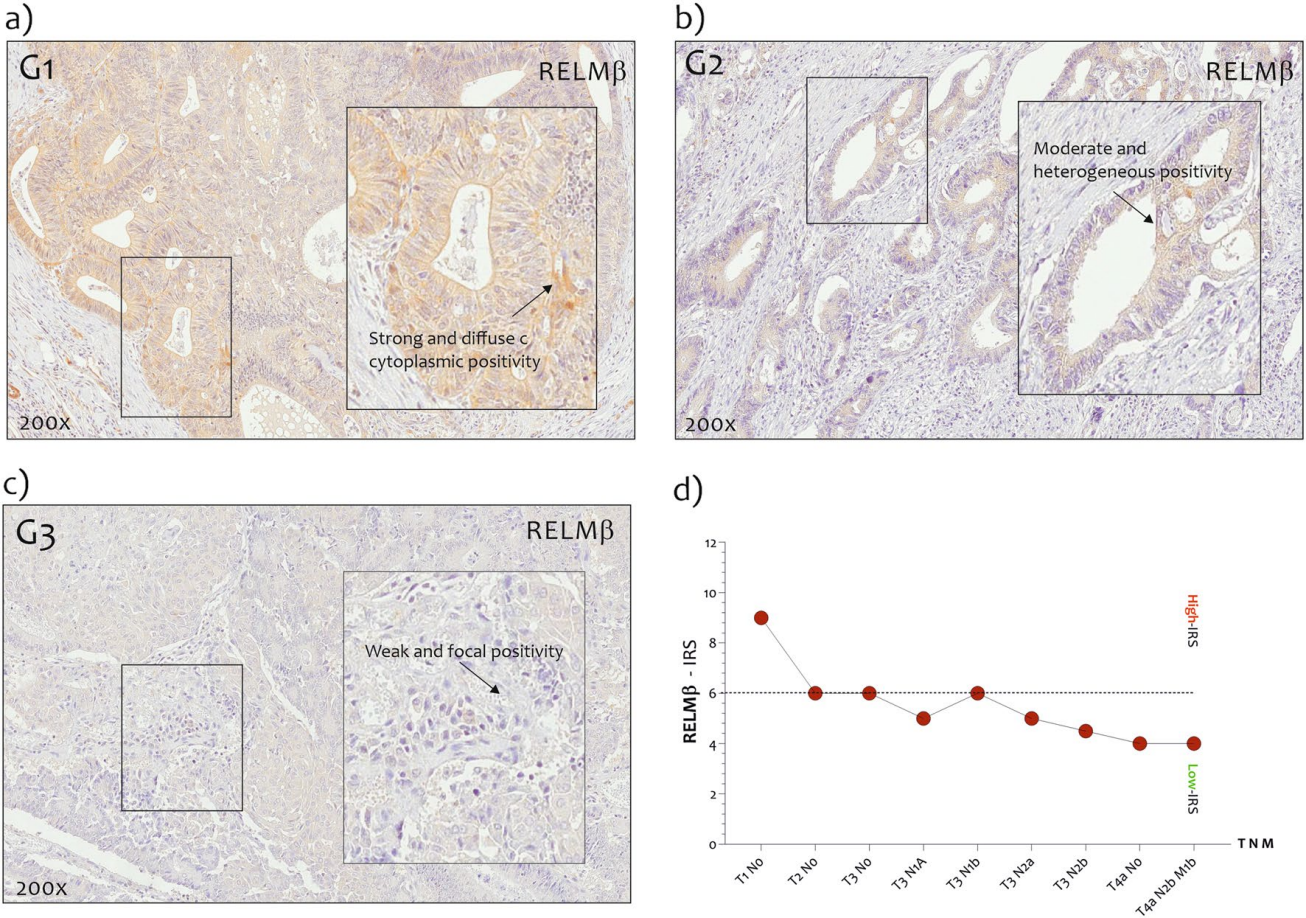
*RETNLB protein detection in CRC biopsies from G1, G2, and G3 grade.* (**a**) Immunostaining of *RETNLB* (RELMβ) in G1 CRC biopsies; (**b**) in G2 CRC biopsies (**c**), and G3 CRC biopsies. The boxes represent magnification of the original figure. The black arrows indicate the location. (**d**) Immunoreactivity Score (IRS) was obtained by multiplying IS and ES and it was considered as low (L-IRS), if < 6, and as high (H-IRS), if ≥ 6, respectively.

In order to confirm our preliminary results obtained silico, we recruited 44 cases of CRC from the Pathology files of the Department “G.F. Ingrassia” of the University of Catania as described in the section Methods, and immunohistochemical analysis was performed. RELMβ positive protein staining was found both in CRC and in healthy colonic mucosa; in detail, positive staining for RELMβ was found, at least focally, in 40/44 (91%) tumors and in 39/44 (89%) portions of healthy colonic mucosa adjacent to the neoplasm and included in the analyzed histologic section. Four out of 44 (9%) tumors were completely negative. The patterns of positivity were: cytoplasmic in 34/40 (85%) tumors and both nuclear and cytoplasmic in 6/40 (15%) tumors. RELMβ L -IRS values were found in 19/44 (43%) tumors; conversely, 21/40 (47%) tumors exhibit RELMβ H-IRS values. As shown in Fig. 7, patients with G1 grade had higher levels of RELMβ compared to G3 patients (Fig. 7a, b). Interestingly, we found a decreasing trend in RELMβ protein levels in CRC biopsies (Fig. 7c). All these finding further confirms our preliminary previously results.

**Figure 8:**
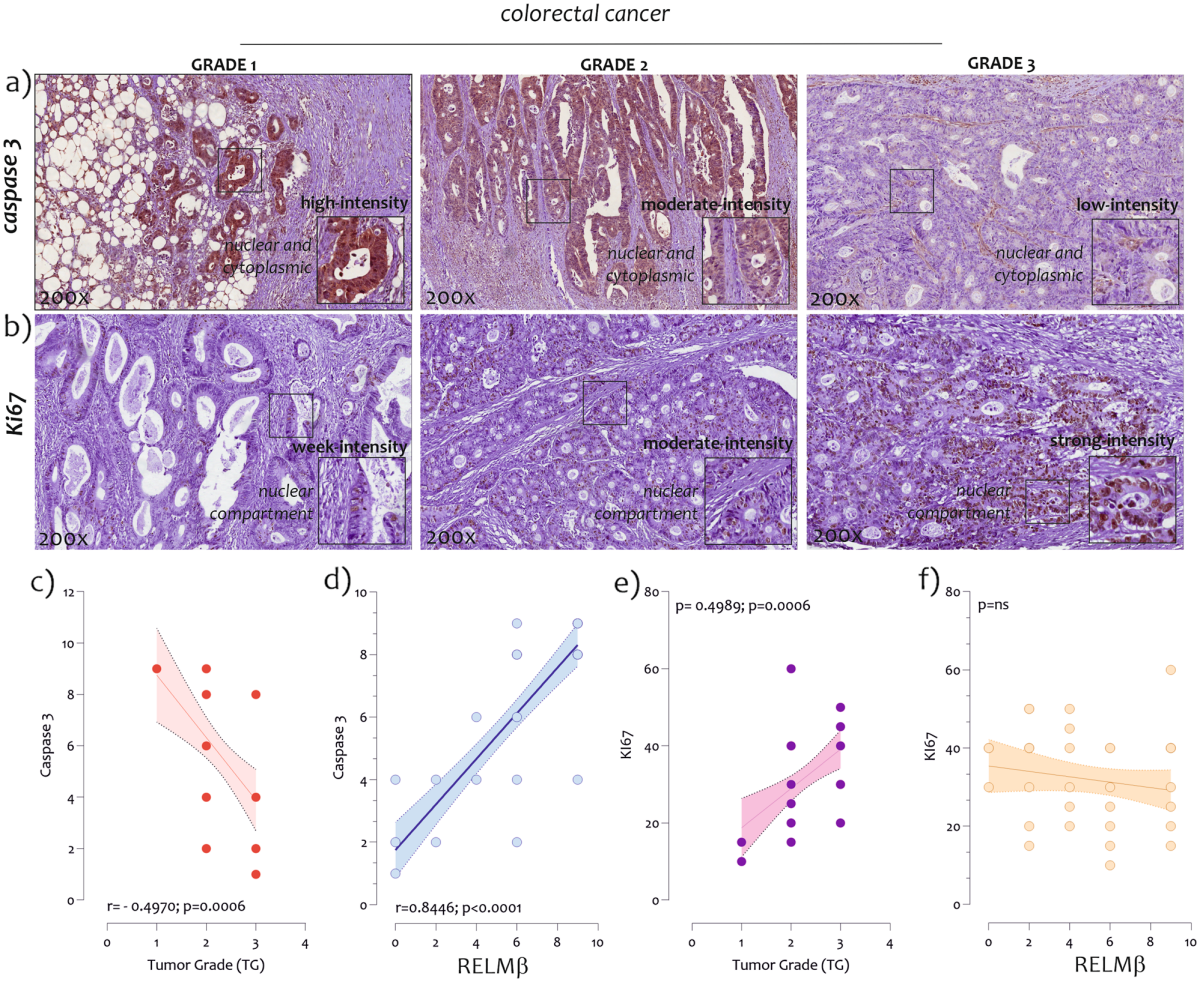
*Caspase 3 and KI67 protein detection in CRC biopsies from G1, G2, and G3 grade.* (**a**) Immunostaining of Caspase 3 in G1, G2, and G3 CRC biopsies; (**b**) Immunostaining of KI67 3 in G1, G2, and G3 CRC biopsies; (**c**) Caspase 3 and tumor grading correlation; (**d**) Caspase 3 and RELMβ correlation; (**e**) KI67 and tumor grading correlation; (**f**) KI67 and RELMβ correlation. The boxes represent magnification of the original figure. Immunoreactivity Score (IRS) was obtained by multiplying IS and ES and it was considered as low (L-IRS), if < 6, and as high (H-IRS), if ≥ 6, respectively.

In order to verify a correlation between RELMβ expression and the main cellular regulatory processes in CRC, we performed an immunohistochemical analysis of the Caspase 3 expression (a major mediator of apoptosis)^14^ and KI67 (a marker of cell proliferation)^15^. Positive staining was found, at least focally, in 44/44 (100%) tumors for Caspase 3, and for KI67 (100%) included in the analyzed histologic section. No tumors were completely negative. As shown in Fig. 8, patients with G1 grade had higher levels of Caspase 3 compared to G3 patients (Figs. 8a), conversely, patients with G3 grade had higher levels of KI67 compared to G1 patients (Fig. 8b). Furthermore, we found a strong negative correlation between Caspase 3 protein levels and tumor grading in CRC biopsies (r = −0.4979; p = 0.0006) (Fig. 8c). A strong positive correlation was highlighted between the Caspase 3 and RELMβ protein levels (r = 0.8446; p < 0.00001) (Fig. 8d). Opposite results were obtained by correlating KI67 expression levels and tumor grade (r = 0.4989, p = 0.0006) (Fig. 8e). No significant correlation was found between KI67 and RELMβ expression levels (Fig. 8f).

## Discussion

The onset and development of CRC is a multifactorial pathology, however, the specific molecular mechanisms underlying its development are not yet fully understood. In recent years, several mutational markers have been identified that have given new information on the etiopathogenesis of this disease. Therefore, the identification of new markers that can support new therapeutic strategies in order to provide new, more effective therapeutic targets is of great scientific interest. Herein, we have examined the expression profile of *RETNLB* gene in CRC. In particular, we analysed four microarray datasets generated from colon biopsies of healthy subjects and CRC patients. We reported that *RETNLB* expression levels were significantly reduced in CRC biopsies compared to healthy and to NPDM. Furthermore, the reduction was significantly correlated with the TNM grade and age of the CRC patients and with mutated subjects for KRAS, TP53, and BRAF. Indeed, lower levels of *RETNLB* levels identified patients with poor prognosis at 5 years, as demonstrated by good separation on Kaplan–Meier and ROC curve. The data obtained from the in-silico analysis were further confirmed by the histochemical analysis carried out both through the Human Protein Atlas portal and by a cohort of 44 patients with CRC recruited for this study.

In recent years, the use of datasets available in public databases has grown exponentially. We and others have extensively used the analysis of public transcriptomes datasets for the identification of novel pathogenic pathways and therapeutic targets in several human pathologies^16–20^ including, neurodegenerative disease^17,21,22^ and cancer^23^. Such experimental approach allow to increase the statistical power to obtain a more precise estimate of gene expression differentials, and assess the heterogeneity of the overall estimate. Meta-analysis offers also the advantage of being relatively inexpensive since it makes comprehensive use of already available data and represents a vast source of information that could make a difference in setting up highly targeted experimental strategies.

*RETNLB* is a colon and small intestine-specific cysteine-rich protein, highly produced by goblet cells specifically in the colon and is secreted apically in the colonic lumen^12^. During mouse embryogenesis, *RETNLB* gene expression is not detected until day 17^10^ because of the remodeling phenomena occurring in the colon including the transition from an undifferentiated stratified epithelium to a simple columnar epithelium^10^. The biological function of *RETNLB* is still elusive and controversial, but its expression seems to be linked to inflammatory phenomena triggered by bacterial infections^24^. Its expression in the gastrointestinal tract may influence the microbial environment and is likely to play a role in defense against parasites and intestinal nematode infections^12^. Recently, it has been highlighted new evidence suggesting the involvement of *RETNLB* in carcinogenesis. Indeed, *RETNLB* is expressed in the metaplastic epithelium of Barrett’s esophagus and enhanced in dysplasia, which can be served as a potential biomarker for such condition^25^. Furthermore, Helicobacter pylori (H. pylori) was reported to be associated with gastric carcinogenesis and infection facilitated the expression of *RETNLB*^24^. It has been shown that overexpression of *RETNLB* could facilitate invasion and migration of gastric carcinoma cells and it increases the expression of Epithelial-mesenchymal transition (EMT) -related proteins, such as N-cadherin, Snail, Vimentin, decreasing the E-cadherin level, promoting the progression of EMT. It is noteworthy that in gastric carcinoma, *RETNLB* levels are inversely related to patient survival^26^.

The *RETNLB* expression levels stratification in CRC patient's biopsies (n = 1211) according to age showed a significant negative correlation, pointing out that *RETNLB* levels decreased with increasing age. Assuming a protective role played by *RETNLB* for the colonic mucosa, the reduction of its levels as a function of age would indicate that with aging the risk of developing cancer increases^27^. Indeed, the positive correlation between the incidence of colorectal cancer and age is a condition largely investigated. Unfortunately, most likely due to the small number of healthy subjects collected (n = 50) for this study, we have not highlighted a significant reduction in *RETNLB* levels as according to of age.

Our results are partially confirmed by bibliographic data present at this time in the literature. In two recent publications, *RETNLB* levels were shown to be inversely correlated with survival in CRC and oral squamous cell carcinoma patients (high *RETNLB* levels are related to a poor prognosis)^28^. These results are in accordance with our findings. In 2009, Li-Duan Zheng and colleagues^13^ reported that of the 80 CRC patients studied, 65 (81.25%) tested positive for RELMβ, mainly in the cytoplasm of colon mucosa. Contrasting sharply with the strongly RELMβ-positive tumors, normal colon mucous membrane was negative or weakly positive. This finding was partially in contrast to their successive observation showing that the mean postoperative survival time (2.76 years) of RELMβ-positive patients was significantly longer than that (1.26 years) of RELMβ-negative patients (p = 0.032). One possible explanation for this apparent discrepant result could be the different distribution of *RETNLB* between the distal and proximal colon. As such, a correct comparison of the districts analyzed could respond to the discrepancy with the results obtained by Li-Duan Zheng and colleagues. In fact, in our results this difference was highlighted in the histochemical analysis using HPA in which we highlighted a greater expression of RELMβ in the rectum compared to the colon. Furthermore, Fuijo J. et al. showed that *RETNLB* expression in the intestine was markedly suppressed by the high-protein and high-carbohydrate diets and therefore the significant differences of our population compared to Chinese patients may be also responsible of different protein expression levels^29^.

Our analysis included the comparison between the *RETNLB* expression levels both in CRC patients wild-type and mutated for the TP53, BRAF, and KRAS genes. We showed that there was a correlation between *RETNLB* expression levels and the mutation status of CRC patients for the genes TP53, BRAF, and KRAS. In particular, the expression levels of *RETNLB* were found to decrease significantly in subjects mutated for the TP53 and BRAF genes, while they increased in subjects mutated for KRAS, all compared to wild-type CRC patients. The role of the tumor suppressor p53, which is the product of the TP53 gene, has been widely demonstrated^30^. The protein is involved in cell cycle arrest, senescence, or apoptosis under cellular stress, as a consequence of DNA damage, hypoxia, and nutrient depletion. TP53 variations have been reported to be correlated with the poor prognosis of 43% of patients with CRC. The reduction of *RETNLB* in TP53 mutant subjects could indicate that in these subjects the loss of function of TP53 could also involve the *RETNLB* transcriptional pathway. As for BRAF mutations (the most frequent is BRAFV600E corresponding to almost 95%), they are considered an unfavorable prognostic factor^31^ in patients with metastatic colorectal cancer (mCRC) and are estimated around 8–12% of patients with mCRC^32,33^. The B-Raf protein is involved in signal pathways involved in directing cell growth. This protein plays a role in regulating the MAP kinase/ERKs signaling pathway, which affects cell division, differentiation, and secretion^34^. The presence of the BRAF V600E mutation in CRC patients could alter the *RETLMB* pathway compromising transcription and consequently biological function. Even though BRAF and KRAS work close in the EGFR pathway, their mutations result in different gene expression patterns, with an even greater heterogeneity found among BRAF-mutated mCRC. KRAS plays an important regulatory role in the cell signal transduction pathways. In CRC, KRAS mutations lead to abnormal activation of it RAS/RAF/MEK/ERK signaling pathway, upregulating IGF-1R expression via a novel MEK-SP1-DNMT1-miR-137 pathway in CRC cells to promote liver metastasis^35^. The KRAS mutation also affects the tumor microenvironment. KRAS mutations are associated with Th1/cytotoxic immunosuppression in CRC. A significant increase in neutrophils in the bloodstream of CRC patients harboring KRAS mutations has been shown to result in increased neutrophil mobilization and tumor site recruitment^36^. This may partly explain the increase of *RETNLB* in KRAS-mutated subjects. Among the biological functions attributable to the increase of *RETNLB* expression is the immunological action against parasites and helminths^37^. Furthermore, an increase in tumor-associated M2-macrophages (typically also activated against parasites) is present in KRAS mutant CRC compared to wild-type KRAS CRC^38,39^.

Moreover, we showed that adjuvant chemotherapy treatment in CRC patients significantly reduced *RETNLB* expression. Surgical resection is the only curative treatment for CRC. The adjuvant chemotherapy is a strategy to eradicate micro-metastatic disease and improve survival. This has been most clearly demonstrated in stage III (node-positive) disease, whereas the benefit of adjuvant chemotherapy in stage II disease remains controversial^40^. Only 30% at the most will actually benefit from this adjuvant treatment, 50% of them being already cured by the surgery and 20% of them experiencing disease recurrence despite the adjuvant treatment^41^. Emerging evidence suggests that gut microbiota may influence the response to chemotherapy. Furthermore, it should be borne in mind that chemotherapy such as 5-Fluorouracil (5-FU) often causes several drawbacks including weight loss, diarrhea, myelosuppression, and intestinal mucositis^42^. All of these events represent a conformational change of the cellular structure of the mucosal epithelium that could interfere with the expression of *RETNLB*. Furthermore, the alteration of the microbiota by the chemotherapy treatment could be closely related to the reduction of *RETNLB*. It has been shown that *RETNLB* is a bactericidal protein that limits contact between Gram-negative bacteria and the colonic epithelial surface. The RELM family is a unique family of bactericidal proteins, of which *RETNLB* plays a role on promote host-bacterial mutualism by regulating the spatial segregation between the microbiota and the intestinal epithelium^43^. Reduction of *RETNLB* could explain microbial dysbiosis and all related downstream events such as bowel alterations.

In addition, the reduction in *RETNLB* expression levels that we highlighted in our investigation could be related to the functional/cytological change of epithelial cells in CRC. In addition, methylation / acetylation phenomena may underlie the reduced levels of *RETNLB*. Our hypothesis is that in some CRC phenotypes, a mutated *RETNLB* phenotype may be present. Currently, polymorphisms for the *RETNLB* gene have not yet been described.

Our finding showed that substantial differences exist in colon *RETNLB* transcript during CRC progression thus suggesting its role as possible new target protein for CRC patients’ prognosis. Interestingly, adjuvant therapy significantly reduces *RETNLB* and such evidence deserves further investigations in order to suggest this protein also as a potential therapeutic target in CRC patients.

## Methods

### Dataset selection

The NCBI Gene Expression Omnibus (GEO) database (http://www.ncbi.nlm.nih.gov/geo/) (Clough and Barrett, 2016) was used to select transcriptomes datasets of interest. Mesh terms “colon”, “human”, and “colorectal”, were used to identify the datasets. We sorted the datasets by the number of samples (High to Low), age and sex of the participants and by the clinical data made available by the authors. Four datasets were selected following the criteria exposed in the section “Clinical and pathological criteria”. The dataset selected are showed in Supplementary Table 1.

### Data processing and experimental design

To process and identify Significantly Different Expressed Genes (SDEG) within the datasets, we used the MultiExperiment Viewer (MeV) software (The Institute for Genomic Research (TIGR), J. Craig Venter Institute, La Jolla, USA). In cases where multiple genes probes have insisted on the same GeneID NCBI, we used those with the highest variance.

### Clinical and pathological criteria of datasets

Most of the samples analyzed were obtained from public tissue banks (Supplementary Table 1). Sample pH, and RNA integrity number (RIN) were elements of pre-selection by the authors of the reference microarray datasets, and subsequently, object to our further exclusion analysis. All patients gave informed consent. This study was performed in accordance with all relevant guidelines and regulations, particularly with the Declaration of Helsinki and its later amendments. The local Institutional Review Board (Catania 1 Ethics Committee, Santa Sofia 78 street, Catania, Italy) reviewed and approved this study. The Ethics Committee at the University of Catania waived the informed consent.

All specimens were derived from primary carcinomas and were snap frozen in liquid nitrogen immediately after surgery for storage at − 80 °C.

### Clinical and pathological criteria of biopsies for immunohistochemistry analysis

All cases of surgically-resected and histologically-proven CRC adenocarcinomas, diagnosed between January 2005 and April 2010, were retrieved from the Pathology files of the Department “G.F. Ingrassia” of the University of Catania. Among these, 44 cases, for which the follow-up data and the paraffin-embedded blocks for additional immunohistochemical sections were available, were selected and included in this retrospective study. For all cases, the clinico-pathologic data were collected from the original pathologic report. Patient and tumor characteristics are summarized in Supplementary Table 1.

### Immunohistochemistry and evaluation of the immunohistochemical results

Immunohistochemical analyses were performed as previously described^44^ after the standard deparaffinization treatments, sections were incubated with anti-*RETNLB*. Negative control slides were obtained by omitting the primary antibodies. The immunohistochemical features of the cases from our series were semi-quantitatively evaluated by two pathologists (G.B. and R.C.), blinded of the clinic-pathologic data, as previously described^45^. Briefly, the Intensity of Staining (IS) and the Extent Score (ES) were evaluated on a scale from 0 to 3 and from 0 to 4, respectively; Immunoreactivity Score (IRS) was obtained by multiplying IS and ES and it was considered as low (L-IRS), if < 6, and as high (H-IRS), if ≥ 6, respectively.

### Data processing and experimental design

In order to process and identify Significantly Different Expressed Genes (SDEG) in all selected datasets, we used the MultiExperiment Viewer (MeV) software (The Institute for Genomic Research (TIGR), J. Craig Venter Institute, USA)^46^. In cases where multiple genes probes insisted on the same GeneID, we used those with the highest variance. The significance threshold level for all data sets was p < 0.05. Statistically significant genes were selected for further analysis. For all datasets we performed a Benjamini & Hochberg FDR (False discovery rate) to adjust P values for multiple comparisons^47–49^.

### The Immunohistochemistry (IHC) using The Human Atlas project

The analysis of data from microarray, provided results about the mRNA expression levels. To confirm these results, we decided to use the web utility “The Human Protein Atlas” (https://www.proteinatlas.org/).^50–52^ This web site is licensed under the Creative Commons Attribution-Share Alike 3.0 International License (https://creativecommons.org/licenses/by-sa/3.0/) for all copyrightable parts (https://wiki.creativecommons.org/wiki/Data) of the database. We selected the immunohistochemistry analysis available on the web site. For each antibody, the observed staining is assigned a validation score. The validation score is based on the result of two different validations that are separately evaluated. The different levels of validation score are Supported, Approved or Uncertain (https://www.proteinatlas.org/about/antibody+validation#ifv). Annotation parameters include an evaluation of staining intensity (SI) (negative, weak, moderate or strong), fraction of stained cells (rare, < 25%, 25–75% or > 75%) and subcellular localization (nuclear and/or cytoplasmic/membranous). Details of analysis performed are available in Supplementary Table 1.

### Statistical analysis

For statistical analysis, Prism 7 software (GraphPad Software, La Jolla, CA, USA) was used. Significant differences between groups were assessed using the Ordinary one-way ANOVA test, and Tukey’s multiple comparisons test correction was performed to compare data between all groups. Correlations were determined using Pearson correlation. All tests were two-sided and significance was determined at adjusted p value 0.05. All datasets selected were transformed for the analysis in Z-score intensity signal. Z score is constructed by taking the ratio of weighted mean difference and combined standard deviation according to Box and Tiao (1992)^53^. The application of a classical method of data normalization, z-score transformation, provides a way of standardizing data across a wide range of experiments and allows the comparison of microarray data independent of the original hybridization intensities. The z-score it is considered a reliable procedure for this type of analysis and can be considered a state-of-the-art methods, as demonstrated by the numerous bibliography^54–65^.

The efficiency of *RETNLB* was assessed by the receiver operating characteristic (ROC) curve analyses. Nonparametric ROC curves analyzed CRC vs healthy. The area under the ROC curve (AUC) and its 95% confidence interval (95% CI) indicates diagnostic efficiency. The accuracy of the test with the percent error is reported^66^. Survival plots based on Kaplan–Meier estimates were generated for cumulative survival (probability of clinical success) and Mantel-Cox proportional hazard ratio calculated. A p < 0.05 was considered statistically significant.

## Supplementary Information

Supplementary Tables.

## Data Availability

The datasets used and/or analysed during the current study available from the corresponding author on reasonable request.
